# DynSig: Modelling Dynamic Signaling Alterations along Gene Pathways for Identifying Differential Pathways

**DOI:** 10.3390/genes9070323

**Published:** 2018-06-27

**Authors:** Ming Shi, Yanwen Chong, Weiming Shen, Xin-Ping Xie, Hong-Qiang Wang

**Affiliations:** 1 State Key Laboratory of Information Engineering in Surveying, Mapping and Remote Sensing, Wuhan University, 129 Luoyu Road, Wuhan 430079, China; shiming@whu.edu.cn (M.S.); ywchong@whu.edu.cn (Y.C.); shenwm@whu.edu.cn (W.S.); 2 Machine Intelligence & Computational Biology Lab., Institute of Intelligent Machines, Chinese Academy of Science, P.O. Box 1130, Hefei 230031, China; 3 School of Mathematics and Physics, Anhui Jianzhu University, Hefei 230022, China

**Keywords:** high-throughput data, pathway analysis, Markov chain model, gene links, dynamic signaling

## Abstract

Although a number of methods have been proposed for identifying differentially expressed pathways (DEPs), few efforts consider the dynamic components of pathway networks, i.e., gene links. We here propose a signaling dynamics detection method for identification of DEPs, DynSig, which detects the molecular signaling changes in cancerous cells along pathway topology. Specifically, DynSig relies on gene links, instead of gene nodes, in pathways, and models the dynamic behavior of pathways based on Markov chain model (MCM). By incorporating the dynamics of molecular signaling, DynSig allows for an in-depth characterization of pathway activity. To identify DEPs, a novel statistic of activity alteration of pathways was formulated as an overall signaling perturbation score between sample classes. Experimental results on both simulation and real-world datasets demonstrate the effectiveness and efficiency of the proposed method in identifying differential pathways.

## 1. Introduction

With the rapid development of high-throughput technology, including microarrays and deep sequencing, tremendous amounts of various omics data have been generated and accumulated, which provides unprecedented opportunities for understanding molecular mechanisms of cells and disease etiology [1,2,3,4,5]. Relative to basic gene units, pathways are higher-level functional modules in cells, and play more critical roles in various cellular processes, such as tumorigenesis [6]. Identifying differentially expressed genes (DEGs) often suffers from being less statistically reproducible and less biologically interpretable, while identifying differentially expressed pathways (DEPs) provides more consistent and more reliable knowledge about cancer or other diseases through maximizing the potential of omics data [7,8]. Currently, computational methods for identifying DEPs is still underdeveloped theoretically and practically, although a large number of computational methods are available for DEG identification [6].

Current methods for identifying DEPs can be categorized into three generations [9]: overrepresentation analysis, functional class scoring, and pathway topology. Overrepresentation analysis methods only test how significantly a pathway contains DEGs than by chance, and employ Fisher’s exact test or geometric distribution-based test to estimate the significance. Functional class scoring methods assume that not only large changes, but also weaker, coordinated changes of genes in pathways can have significant effects on pathway activity. The gene-level statistics for all genes in a pathway are aggregated into single pathway-level statistics for the significance test. Generally, the first two generations have two main shortcomings: (1) equally treating all the genes in a pathway and (2) ignoring topological information, which also likely dominates the activity of pathways. For example, many overrepresentation analysis methods rely on comparing the numbers of DEGs between pathways and a given reference gene list [10,11]. These methods are often overdependent on the division of DEGs and non-DEGs that is hard to pre-define in practice. To overcome the situation, Subramanian et al. [12] resorted to ranking genes according to their associations with the phenotype, instead of the division as usual, and presented a second-generation method, gene set enrichment analysis (GSEA). Many variants of GSEA have so far been proposed, which, including GSEA, generally follow three fundamental steps: (1) estimating the association of each gene’s expression pattern with phenotype by *t*-statistics or other correlation measures; (2) calculating pathway-level enrichment evidence score based on the association scores; (3) determining the differential significance of pathways using empirical distribution or permutation test. Others rely on the fitness of regressing phenotypes on pathway member genes for DEP identification, e.g., Global test by Geoman et al. [13] and ScorePAGE by Rahnenfuhrer et al. [14]. Due to the ignorance of pathway topology, these second-generation methods tend to fail in real-world applications, often producing same or similar results for pathways that have identical or similar member genes [9].

Recent studies evidenced that the third-generation pathway topology methods can considerably improve pathway analysis in practice, by incorporating pathway topological information. To extract pathway topological information, Gao and Wang [15] introduced the concept of molecular connectivity from chemoinformatics (TAPPA). By mimicking molecular connectivity, TAPPA defines a pathway connectivity index based on gene connectivity, and employs the Mann–Whitney test to compare pathway connectivity indexes between two classes for DEP identification. Isci et al. [16] introduced Bayesian networks to infer pathway activity (BPA) for pathway analysis. In the BPA, a pathway is first simplified into a directed acyclic graph, and is modeled as a Bayesian network to score the degree to which expression data fit the pathway topology. BPA can preserve the dependencies’ underlying pathway topology, and lead to an improvement of characterizing pathway activity. Other researchers considered transforming or encoding pathway structures into a few principal components, and combining with gene-level statistics for pathway analysis. For example, Jacob et al. [17] applied spectral analysis to Fourier-transform a pathway graph (DEGraph). DEGraph can detect the change of pathway expression between sample classes in terms of low frequency components from a pathway spectrum. From a networking perspective, a pathway is essentially comprised of well-organized gene links along which biological signals pass, informing cellular activity. Genes are ubiquitous, connecting each other in pathways, and the interactions are fundamental to maintain the activity of a biological system. Therefore, it is necessary and crucial to mine and utilize information on gene links for characterizing DEPs in cancer research [18,19,20].

In this paper, we propose to model gene links to capture biological signaling along them, and detect the dynamic alteration of pathway topology for identifying DEPs (DynSig). Biologically, gene links can represent various types of gene associations, e.g., binding, inhibition, or activation, and they may be activated or silenced for a biological function in a particular cellular state. Recently, Han et al. [21] presented an edge set enrichment analysis, which follows the idea of GSEA to only measure the enrichment of informative edges for identifying DEPs. We, here, employ the Markov chain model (MCM) to model gene links where biological signals are passed from the source gene to the destination gene. DynSig characterizes the state transition between any two linked genes, and can directly make inference about the activity of the pathway composed of the gene links. To identify DEPs, a new pathway-level statistic that accounts for the accumulated dynamic alteration of pathway between classes is proposed. In experiments, we evaluated the proposed method on both simulation data and real-world expression data, and compared it with previous methods. Experimental results demonstrate the effectiveness and efficiency of the proposed method in identifying differential pathways.

## 2. Materials and Methods

### 2.1. Framework of DynSig for Pathway Analysis

**Figure 1:**
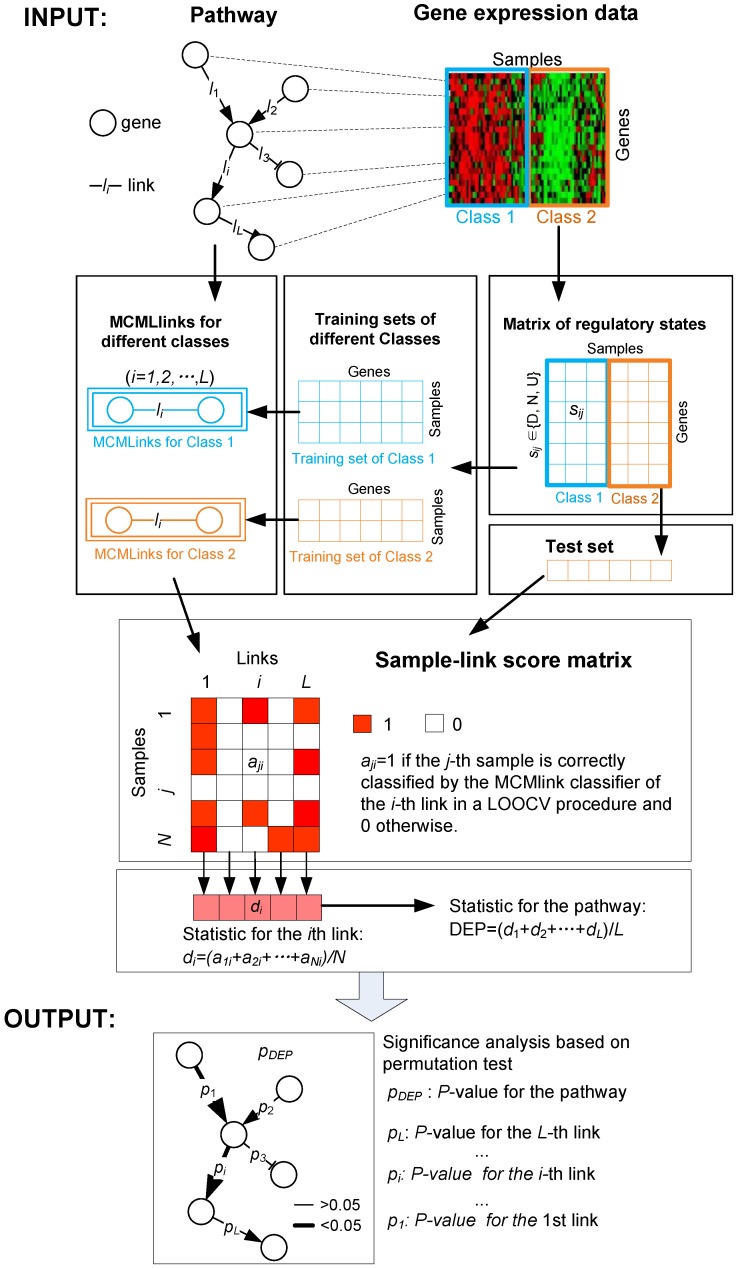
The flowchart of the proposed method DynSig. The raw input data includes an *L*-link pathway and a continuous expression matrix with sample labels (e.g., *Class 1* vs. *Class 2*). The continuous gene expression values are discretized into three regulatory states based on discretization methods: downregulated (D), non-regulated (N) and upregulated (U). Furthermore, all the samples are divided into three sets: two training sets for *Class 1* and *Class 2*, respectively, and a test set comprising the rest of samples. For the *i*-th link (*i* = 1,2, …, *L*) in the pathway, two Markov chain model links (MCMLinks) are built based on the two training sets, respectively. Then, by using the leave-one-out-cross-validation (LOOCV) procedure, the *j*-th sample is classified by the *i*-th link and we have *a_ji_* = 1 if the classification is right and 0 otherwise. The classification power of the *i*-th link is calculated by averaging the resulted scores {*a_ji_*}*_j_*_=1,2,…,*N*_, i.e., *d_i_ =* (*a_1i_ + a_2i_ +* … *+ a_Ni_*)*/N* and the classification power of the pathway is calculated by averaging the classification powers of all links, DEP = (*d*_1_ + *d*_2_ + … + *d_L_*)/*L*. Permutation test is utilized for evaluating *p*-values of the resulted classification powers.

Pathways as gene networks consist of a set of gene nodes and a set of gene links. The gene links reflect regulatory, signaling, or binding relationships, in which the source gene activates or inhibits the destination gene. From a regulatory aspect, genes could be in one of three regulatory states: downregulated, non-significantly regulated, or upregulated. The state transition patterns along gene links are potentially associated with the distinction between two phenotypes of cells. For example, if they are both overexpressed in cancer tissues relative to those in normal tissues, one can say that the gene links are likely related to, or even drive, cancer. We reason that pathways with many such cancer-related gene links should be associated with cancer, and here, present a gene link-based method (DynSig) for identifying DEPs, in which the disparity of pathways in signaling dynamics between classes is explored and utilized. Figure 1 illustrates the framework of DynSig. Specifically, given a pathway, the method first decomposes it into a set of gene links and introduces Markov chain model to model the signaling pattern along each gene link in each class. Then, each link is scored based on an MCM-based classification rule, via a leave-one-out cross validation procedure, which subsequently results in a sample-link score matrix. Based on the score matrix, DynSig finally statistically detects differentially expressed links and DEPs.

### 2.2. Data Preparation

An increasing number of methods have been developed to discretizate continuous gene expression data for analyzing gene expression patterns. Generally speaking, these methods discretizate the continuous gene expression values into three discrete gene regulatory states: downregulated (D), non-regulated (N) and upregulated (U). Based on the discretization, a matrix of continuous gene expression can be transformed into a matrix of regulatory states, i.e., ${\left\{s_{ij}\right\}}_{M\times N}$, where $s_{ij}\in \{D,N,U\}$ represents the regulatory states of the *i*-th gene in the *j*-th sample, *M* is the number of genes, and *N* is the number of all samples. In this paper, the recently developed biology-constrained method [22] is used as the default method to discretize gene continuous expression data.

Without loss of generality, suppose that all the gene expression samples belong to two classes (e.g., control vs. case or cancer vs. normal). Then these samples can be divided into three sets: Two training sets with samples selected from the two classes, respectively, and a test set that comprises all of the rest samples.

### 2.3. Modeling the Dynamics of Gene Links Using Markov Chain Model

**Figure 2:**
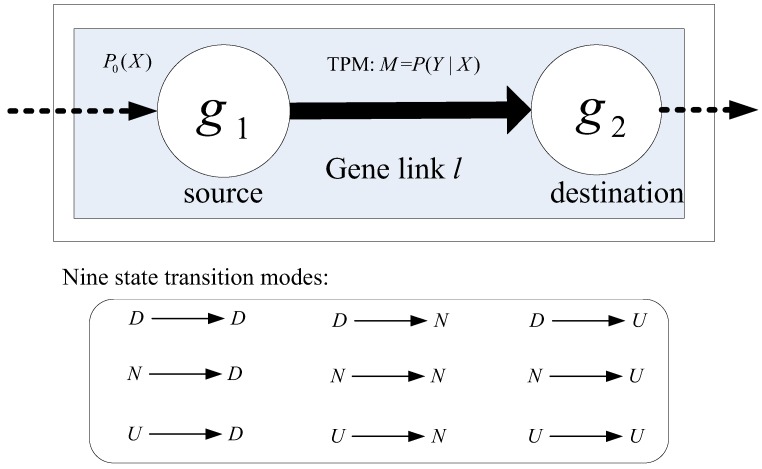
Markov chain model for a gene link (MCMLink). The box above illustrates a MCMLink *l* on a training set. Matrix *M* = *P* (*Y*|*X*) is the state transition probability matrix (TPM) of *l* on the training set. The box below shows all the nine possible regulatory state transition modes, downregulated (D), non-regulated (N) and upregulated (U), delivered by the link *l*.

In statistics, a Markov chain is defined as a stochastic process with Markov property, in which the next state depends only on the current state but irrelevant with any previous event along the chain [23]. Generally, there are two types of Markov chain models, i.e., stationary and non-stationary, which differ in whether transition matrix is shared along the process or not [23]. Compared with stationary MCM, non-stationary one allows different state transition probability matrices (TPMs) for each transition step along the chain of process, so that complex stochastic processes can be modeled without information loss. Considering the complexity of signaling between genes, we here employ non-stationary MCM to model pathways. Specifically, we here consider a chain consisting of two adjacent genes, i.e., gene link, and establish its MCM, which can be readily extended to signaling along a cascade of three or more genes. Figure 2 illustrates the MCM of gene links (MCMLink). Topologically, a pathway consists of a set of gene links comprised of two adjacent genes. We view a gene link *l* as a Markov chain of two genes (nodes), which transits biological information from the source gene *g*_1_ to the destination gene *g*_2_, as shown in Figure 2.

Mathematically, an MCM can be specified by three parameters, i.e., a finite set of discrete states, initial probability distribution of states (*P*_0_), and a state transition probability matrix *M*, as shown in Figure 2. From a viewpoint of biology, we simply specify a tri-state set {D, N, U} for nodes within an MCM. Given continuous gene expression values, we infer the regulatory states of a gene using discretization methods, for example, the recently developed biology-constrained method [22]. Along a gene link, the regulation states transit to carry signaling specific to a particular biological process. The signaling patterns beneath the gene link are encapsulated in the initial state distribution and the state transition probability matrix. We need to learn them from training data as follows. 

Given a gene link *l* from *g*_1_ to *g*_2_ and a *N_train_*-sample training set for class *control*/*case*, we denote the initial state distribution as *P*_0_(*x*), *x* = D, N, or U for the source gene *g*_1_ and the TPM as *M* = {*m_ij_*, *i*, *j* = 1, 2, 3}, where *m_ij_* represents the regulatory state transition probability from the *i*-th state on gene *g*_1_ to the *j*-th state on gene *g*_2_. Totally, there are nine state transition modes, as shown in Figure 2. The initial distribution *P*_0_(*x*) is estimated as the likelihood of gene *g*_1_ being in state *x—*in other words, the frequency of gene *g*_1_ being in state *x* in the training set, is

(1) $$P_{0}\left(x\right)=\frac{1}{N_{train}}\sum_{j=1}^{N_{train}}I\left(s_{g_{1},j},\;x\right),$$

where $s_{g_{1},j}$ represents the state of gene *g*_1_ in the *j*-th sample in the training set, and *I*(·,·) is an indicator function, yielding 1 if the two elements are equal and 0 otherwise. Similarly, we estimate *m_ij_* in M as follows: Let *x* and *y* represent the *i*-th and *j*-th states in the set {D, N, U}, respectively. With *g*_1_ as the source gene and *g*_2_ as the destination gene, *m_ij_* (*i*, *j* = 1, 2, 3) that represents the conditional likelihood of *y* occurring on *x* in this *N_train_*-sample training set can be estimated as

(2) $$m_{ij}=P\left(y|x\right)=\frac{\sum_{i=1}^{N_{train}}I\left(s_{g_{1},i},x\right)\&I\left(s_{g_{2},i},y\right)}{\sum_{i=1}^{N_{train}}\sum_{y\in \{D,N,U\}}I\left(s_{g_{1},i},x\right)\&I\left(s_{g_{2},i},y\right)},$$

where $s_{g_{1},i}$ and $s_{g_{2},i}$ are the states of genes *g*_1_ and *g*_2_ in the *i*-th sample in the training set. 

Based on the MCM of *l* on the *N_train_*-sample training set above, a statistical way to infer the class likelihood of an observed chain sample from the test set is provided. Assume a test sample *t*, in which the two genes of the gene link *l*, *g*_1_ and *g*_2_ are in regulatory states *x*′ and *y*′, respectively. The likelihood of the test sample *t* belonging to the same class as the *N_train_*-sample training set can be estimated as a joint probability, i.e.,

(3) $$P(t)=P(x^{\prime },y^{\prime })=P_{0}(x^{\prime })P(y^{\prime }|x^{\prime }),$$

where *P*_0_(*x*′) and *P*(*y*′|*x*′) are the initial probability of *x*′ and the conditional probability of state *y*′ (gene *g*_2_) on state *x*′ (gene *g*_1_) that can be obtained by Equations (1) and (2), respectively.

#### 2.3.1. Scoring Gene Links for the Disparity of Signaling Dynamics

Biologically, gene links can exhibit different activity levels in different cellular states, and so a non-trivial gene link can distinguish two different phenotypes due to the disparity of signaling dynamics. Given two MCMs of a same gene link on training sets of two classes (e.g., *control* and *case*), we can calculate the likelihoods of a test sample *t* belonging to the two classes according to Equation (3) and subsequently, the test sample *t* is assigned to the class with the larger likelihood. Based on the classification rules above, we apply leave-one-out cross–validation (LOOCV) to recursively divide the whole *N* samples into training sets and a test set consisting only one sample. Suppose ${\left\{a_{ij}\right\}}_{N\times L}$ are the overall classification results with *a_ij_* indicating the classification of the *i*-th sample by the *j*-th link, we have *a_ij_* = 1 for correct classification and 0 otherwise. The overall classification power of the *j*-th gene link on all the *N* samples is defined as *d_j_* = (*a*_1*j*_ + *a_2j_* + … + *a_Nj_*)/*N*, where *N* is the total number of samples. 

To assess the statistical significance of the overall classification power of the *j*-th link, i.e., *d_j_*, a permutation test pipeline is then designed: (1) Randomly shuffling the class labels of samples; (2) applying the LOOCV procedure to the shuffled data; (3) repeating (1–2) *B* = 1000 times and obtaining *B* accuracies *rd_b_*, *b* = 1, 2, …, *B*; 4) Calculating the *p*-value for the observed *d_j_* as

(4) $$P_{d_{j}}=\frac{1}{B}\sum_{b=1}^{B}I\left(d_{j}<rd_{b}\right)$$

where *rd_b_* is the *b*th *rd* and *I* is an indicator function yielding 1 if true and 0 otherwise. 

#### 2.3.2. Identifying Differentially Expressed Pathways

Since a pathway is essentially a set of organized gene links, the activity of it can be estimated based on the activity of the gene link components. Assume a pathway that contains *L* gene links. Let *d_j_* represent the score of the *j-*th gene link, a novel statistic for measuring the pathway-level differential expression can be calculated as

(5) $$DEP=\frac{1}{L}\sum_{j=1}^{L}d_{j}.$$

The statistic, *DEP*, reflects the disparity of signaling dynamics in the pathway between two classes. From Equation (5), it can be seen that the calculation of *DEP* only involves the association perturbation of two directly connected genes in the pathway, and thus avoids noise or bias from the expression of individual genes.

To estimate the significance of the DEP score of a pathway, permutation test is utilized here. Specifically, Z = 1000 random pathways are generated with the same genes and the same number of links (i.e., *L*) in the original pathway. Then, the *p*-value for DEP of the original pathway can be calculated as

(6) $$p_{DEP}=\frac{1}{Z}\sum_{i=1}^{Z}I\left(DEP<rDEP_{i}\right),i\in \left\{1,2,\ldots ,Z\right\},$$

where *rDEP_i_, i =*1, 2, *...*, *Z*, is the *DEP* value of the *i*th random pathways. A pipeline of the proposed approach DynSig is shown in Figure 1.

#### 2.3.3. Principal Pattern of Signaling Dynamics Specific to a Cancer Type

Pathways are a directed gene network in which genetic or physiological information flows along gene chains. In a differentially expressed pathway, there may exist principal patterns of signaling dynamics specific to the cancer type. The use of MCM here allows for finding such principal patterns that consist of principal state transitions of consecutive gene links along a cascade gene chain. Considering that each link is modeled individually, we determine the principal pattern of a given cascade chain starting from the ending link that is usually key to the biological role that the chain plays. For the ending link, the principal state transition will be taken as one of the nine modes that has the maximum transition probability. Then, we determine the principal state transition of the penultimate link. Suppose that the destination gene of it is *A*, which is also the source gene of the ending link. The principal state transition is the mode that has the largest transition probability conditioned on the principal state of *A* in the ending link. Similarly, the principal state transitions of other subsequent links along the chain are determined. Finally, we obtain the principal pattern of the chain by concatenating the resulting principal state transitions of each link.

### 2.4. Simulation Data Generation

**Figure 3:**
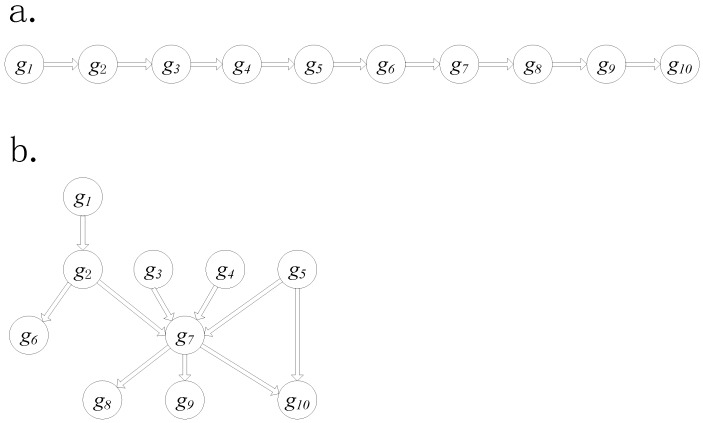
Two types of pathway structures: (**a**) the cascade structure, and (**b**) the complex structure used in simulation data.

Assume two classes (control vs. case) of equal sizes *n* = 60 and 200 pathways, of which one half are DEPs that are differentially expressed between the two classes, and the other half are non-DEPs The 200 pathways follow two types of pathway structures: a cascade structure consisting of ten genes, and a complex structure mimicking a real Kyoto Encyclopedia of Genes and Genome (KEGG) pathway (cell cycle), as illustrated in Figure 3. Three regulatory states of genes are assumed, i.e., *S* = {D, N, U}, where D, N, and U represent downregulated, non-regulated, and upregulated, respectively. For a sample class, genes associated with it are assumed to be predominantly in one of the three regulatory states (major state), and pathways associated with it then exhibit principal state transition patterns consisting of the major states of such genes. Accordingly, each of the 100 DEPs were generated to take two different principal patterns of activity in the case and control classes respectively, while each of the 100 non-DEPs were assumed to be in a same principal pattern between the case and control classes. Let *r* be the probability of the dominant pattern of a pathway, the major states of the associated genes will be present at a probability of *r*, and the two other states are preset to occur at an equal odd of (1 − *r*)/2. From a viewpoint of biology, the probability (*r*) reflects the variability of cell systems under a particular phenotype. Gene expression values are then simulated as follows: When genes are in major states, their expression values were randomly sampled from normal distributions, *N* (1, 4), *N* (3, 4), and *N* (5, 4), for state D, N, and U, respectively, and when genes are in minor states, the expression values from different gamma distributions, Γ (1, 0.5), Γ (3, 0.5), and Γ (5, 0.5), for state D, N, and U, respectively. Additionally, to examine the effect of DEGs on DEP identification, we also varied the proportion (ρ) of DEGs in DEPs when generating the simulation data. In summary, we totally simulated 2 × 3 × 4 data scenarios by varying *r* = {50%, 60%, 70%, 80%} and ρ = {30%, 50%, 70%} for the two types of pathway structures, and generated 20 random datasets in each data scenario.

## 3. Results

### 3.1. Simulation Data Study

We first applied the proposed method to analyze the simulated data. For each simulation scenario, we calculated (i) average accuracy (ACC) of DEP identification, (ii) true positive rate (TPR), (iii) false positive rate (FPR), (iv) positive predictive value (PPV), (v) false negative rate (FNR), (vi) Matthews correlation coefficient (MCC), and area under the curve (AUC) of receiver operating characteristics over 20 random datasets of same scenarios. Mathematically, these measures are defined as

(7) ACC=(TP+TN)/(TP+TN+FN+FP),

(8) TPR=TP/(TP+FN),

(9) FPR=FP/(FP+TN),

(10) PPV=TP/(TP+FP),

(11) FNR=FN/(FN+TP),

(12) $$MCC=\frac{\left(TP\times TN-FP\times FN\right)}{\sqrt{\left(TP+FP\right)\left(TP+FN\right)\left(TN+FP\right)\left(TN+FN\right)}},$$

where *TP*, *TN*, *FP*, and *FN* denote the numbers of true positives, true negatives, false positives, and false negatives, respectively. For comparison, we also analyzed the simulation data using the previous five methods, Global test [13], LRpath [24], TAPPA [15], Clipper [25], and DEGraph [17]. Global test fits the relationship between gene expression and clinical outcome of samples using a generalized linear model, and calls pathways significantly differentially expressed if any of regression coefficients is statistically non-zero [13]. LRpath relates the odds of a gene belonging to a predefined pathway with the significance of differential expression in a linear function, and then employs a Wald statistic to determine if a pathway is significantly differentially expressed. Compared with Global test and LRpath, the three methods, TAPPA, Clipper, and DEGraph, can make use of the information of pathway topology for the identification of DEPs. For example, similar to our method, TAPPA is based on gene links, but uses a pathway connection index as a differential expression statistic, and Clipper models gene expression distributions of different sample groups with different graph Gaussian models of a pathway graph. Comparatively, DEGraph relies on spectral analysis to extract pathway topological information for pathway analysis. Table 1 summarizes the results by the six methods at an ad hoc *p*-value cutoff of 0.05 for different data scenarios of *r* = 0.5 and *ρ* = 0.3, 0.5, or 0.7 for the two types of pathway structures. From this table, we can clearly see that our method outperforms all the previous methods with highest ACC, PPV, MCC, and AUC values in almost all data scenarios, showing the superior performance of the proposed method. Among the five previous methods, LRpath, DEGraph, Clipper, and Global test obtained similar poor results, which are inferior to those by TAPPA. Similar observations were made for other data scenarios of *r* and *ρ* (Tables S1–S3).

Figure S1 illustrates the changes of AUCs with the probability parameter *r* for different methods in differential scenarios of *ρ*. From this figure, it can be found that our method achieved the highest AUCs among these methods in all the scenarios. Even when *r* is lowered to 0.5, i.e., only 50% samples have genes in their major states, the AUCs of our method are still up to more than 80%, whereas small *r* significantly degrades the performance of the previous methods. This suggests that our method is insensitive to noise (*r*), but most of the previous methods not. Figure S1 also reveals that all the methods exhibit an increasing pattern of AUCs with *r* in different *ρ* scenarios, as expected. Further comparison indicates that our method and TAPPA led to very close changing patterns in all the scenarios, which should be because they both are based on gene links. At the same time, three previous methods, Global test, DEgraph and Clipper, had similar changing patterns, which should be because they all are based on the utility of gene correlation via multivariate analysis. Among all these methods, only LRpath considers neither gene correlations nor pathway topology, which should be the reason for its poorest AUC performance, as shown in Figure S1.

### 3.2. Applications to Real-World Expression Data

To evaluate the proposed method on real data, we collected two benchmark gene expression datasets: liver cancer dataset [26] and acute lymphocytic leukemia (ALL) dataset [27]. For the liver cancer data, all the samples are divided into two groups: One in which patients had early intrahepatic recurrence (n1 = 20, REC) and another in which patients did not (n2 = 40, NREC), and each sample consists of the expression levels of 7129 probes. The ALL dataset characterizes gene expression signatures in acute lymphocytic leukemia cells associated with known genotypic abnormalities for adult patients, and consists of n1 = 37 patients with presence of *BCR/ABL* gene rearrangement (BCR group) and n2 = 41 patients with absence of *BCR/ABL* gene rearrangement (NEG group). Each sample in the dataset consists of the expression levels of ~11,556 probes. We preprocessed the two datasets as follows: The intensities of multiple probes matching a same Entrez ID were averaged as the expression values of the gene, and non-specific or noise genes were filtered out using a coefficient of variation (CV) filter [28] with a CV cutoff of 0.05. To apply MCMLink, all the genes in the two datasets were discretized into three states, downregulated (−1), non-regulated (0), and upregulated (1), using the biology-constrained discretization method [29]. 

For the analysis, 220 pathways were downloaded from KEGG database [30] and used as candidate pathways. Since for a given pathway, not all genes in it are present in a particular dataset, we examined the member genes of these pathways with the two datasets. By examination, 213 of the 220 pathways were found to have more than one gene link present in the liver cancer dataset, and 218 have more than one gene link present in the ALL dataset. We considered only these qualified pathways in subsequent analyses on the two datasets.

#### 3.2.1. Identification of Differentially Expressed Pathways

We identified DEPs with DynSig for the two real-world datasets. For each pathway, the obtained *p*-value was adjusted using SLIM [31] to produce a *q*-value for controlling false positive rate (FPR). As a result, for the liver cancer data, 48 pathways (Table S4) were called significantly differentially expressed between the two liver cancer classes at an ad hoc *q*-value cutoff of 0.1, and 102 (Table S5) for the ALL dataset. For comparison, five previous methods, Global test [13], LRpath [24], Clipper [25], TAPPA [15] and DEGraph [17], were also applied to analyze the datasets. Figure S2 shows the cumulative probability distribution (CPD) curves of *q*-values across all the pathways by each of these methods for the two datasets. From Figure S2, it can be found that the CPD curve of our method has a larger increase of CPD, around 0.05, than those of the previous methods, irrespective of whether the data are liver cancer data or the ALL data, suggesting the superior power of our method in identifying DEPs.

For the liver cancer data, three previous methods, Global test, LRpath, and TAPPA called no differentially expressed pathways at an ad hoc *q*-value cutoff of 0.1, and another two previous methods, Clipper and DEGraph, only three and one pathways, respectively. By relaxing a *p*-value cutoff to 0.05, these previous methods still called very few pathways as being significantly differentially expressed: 9 for Global test, 8 for LRpath, 7 for TAPPA, 27 for Clipper, and 14 for DEGraph, respectively. Literature survey shows that most of DEPs identified by our methods were previously reported to be related to liver cancer, for example, p53 signaling pathway (*p*-value = 0.012), transcriptional misregulation in cancer (*p*-value = 0.003), and hepatitis B (*p*-value = 0), which are likely differentially expressed between recurrent and non-recurrent liver cancer. However, these pathways were not called significantly expressed by all the previous methods. 

For the ALL data, the five previous methods led to disparate results: three, LRpath (7), TAPPA (9), and DEGraph (60), called relatively few DEPs at an ad hoc *q*-value cutoff of 0.1, while other two methods, Global test (136) and Clipper (132), called a large number of DEPs. Compared with the previous methods, DynSig called 102 DEPs at an ad hoc *q*-value cutoff of 0.1, which is moderate and may be more reasonable, statistically. Given the presence of the *BCR/ABL* chimera, pathways including *BCR* and/or *ABL1* would be biologically affected, and be true DEPs between the two classes of ALL, BCR, and NEG. Of the total 218 pathways, nine were found to be *BCR* and/or *ABL1*-involved according to KEGG pathway annotation. Table 2 shows the identification results of the nine pathways by DynSig and the five previous methods. From this table, it can be found that TAPPA missed eight of the nine pathways and LRpath found only four. Martini et al. [25] previously reported that GSEA [12], SPIA [32] and BPA [16] called only 2, 2, 1 of the 9 pathways at a uncorrected *p*-value cutoff of 0.1, respectively. Compared with these results, our method recognized almost all the nine BCR/ABL1-related pathways (8), which is comparable with the three previous methods, Global test (8), Clipper (7), and DEgraph (7).

#### 3.2.2. Gene Links Play Significant Roles in Pathway Activity

**Figure 4:**
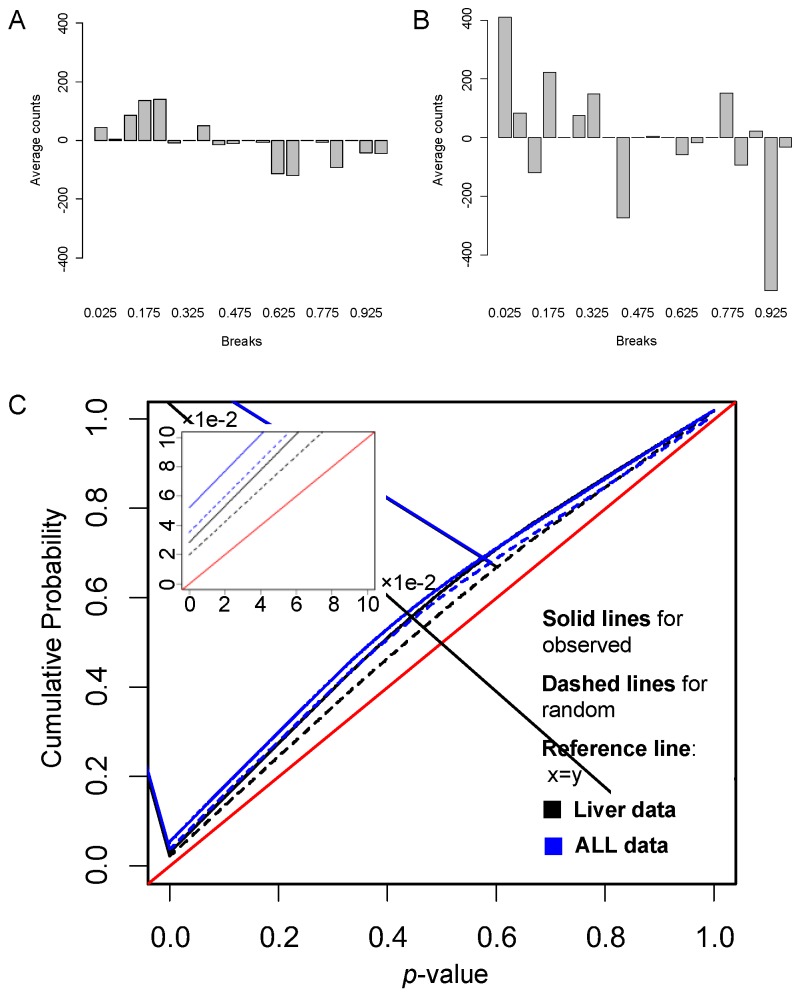
Differences between the *p*-value distributions of true gene links and random links on liver data and ALL data. (**A**,**B**) The differences between counts of *p*-values resulted by the true gene links and the randomly generated links in each bin for (**A**) liver data, (**B**) ALL data, and (**C**) the cumulative probability distributions of *p*-values of true links and random links on liver data and ALL data.

Gene links, as a dynamic element of a pathway, may play crucial roles in pathway activity. We examined how such gene links classifies samples. For the liver cancer data, the resulting 48 significant pathways contain 9949 unique gene links present in the dataset. Figure 4A shows the *p*-values of these links classifying the dataset. For comparison, we randomly sampled the same number of random gene links from the total genes, and calculated their *p*-values of classifying the dataset based on DynSig. Figure 4A illustrates the differences between counts of *p*-values in bins resulted by the true links and randomly generated links on liver data. From this figure, we can clearly see that, compared with the randomly generated links, the true links had more small *p*-values (e.g., <0.5) and fewer large ones (e.g., >0.5), showing that gene links of the selected DEPs are more discriminative than by chance. Similar results were obtained on the ALL dataset (26,309 true gene links), as shown in Figure 4B. We also compare the cumulative probability distributions (CPD) of *p*-values between the true and random gene links for the two datasets, as shown in Figure 4C. When a classifier is not discriminative, the *p*-values will uniformly distribute between 0 and 1, and have a CPD curve along the diagonal line: *y* = *x*. From Figure 4C, we clearly see that, on both datasets, the true gene links hold CPD curves farther away from *y* = *x* than the those of the random links, especially with *p*-values < 0.5. These suggest the better classification power by the true gene links (*p*-value < 2.2×10^-16^ according to a *t*-test). Taken together, these results demonstrate that gene links, as a dynamic element in pathway activity, tend to be discriminative between cancer and normal tissues or between different cancer subtypes.

**Figure 5:**
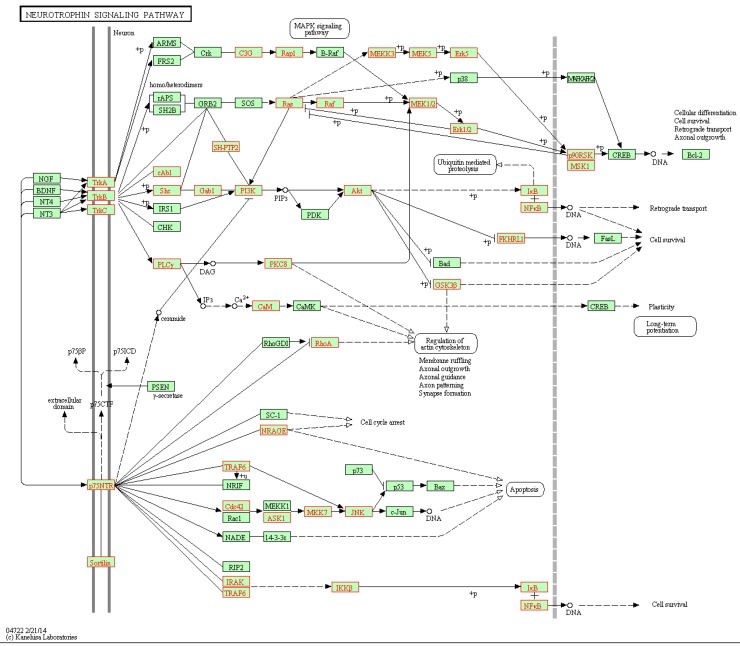
Neurotrophin signaling pathway and genes involved in the significant gene links (marked in red box).

We then overlaid genes involved in the significant gene links (*p*-value < 0.05) onto the network map of pathways. For the ALL data, one of DEPs called by DynSig is neurotrophin signaling pathway (KEGG ID: hsa04722), which as an *ABL1*-involved pathway that has been proven to behave distinctly between BCR and NEG ALL patients. Biologically, neurotrophin signaling transmits positive signals like enhanced survival and growth, and interplays with a variety of intracellular signaling cascades, such as MAPK pathway. Figure 5 shows the overlaid neurotrophin signaling pathway downloaded from KEGG database. It can be seen that hub genes tend to be involved in the significant links, as expected, and that most gene links along the paths towards the biological end of cell survival are called significant to the classification of BCR-ABL and NEG tissues. Among the paths, in particular, two involving nuclear factor-κB (NF-κB) and starting from TrkA/B/C and p75NTR, are exclusively recognized, with all involved gene links significant. 

Biologically, NF-κB as a family of transcription factors regulates the genes involved in inflammatory responses, proliferation and differentiation [33]. It is well known that *BCR-ABL* fusion results in the activation of NF-κB that can trigger tumorigenesis [34,35]. We then examined the state transitions of the link IKBA–NFκB in BCR–ABL and NEG tissues. Results reveals that the state transition with maximum probability is N → U in BCR–ABL patients, but N → N in NEG patients, suggesting that the dissociation between *IKBA* gene and NF-κB is activated in *BCR-ABL*, but not in NEG. The dissociation of *IKBA* from NF-κB in BCR-ABL should activate NF-κB and consequently, cell proliferation. 

#### 3.2.3. Principal Patterns of Pathways Reflect Abnormality of Signaling Dynamics in Cancer

**Figure 6:**
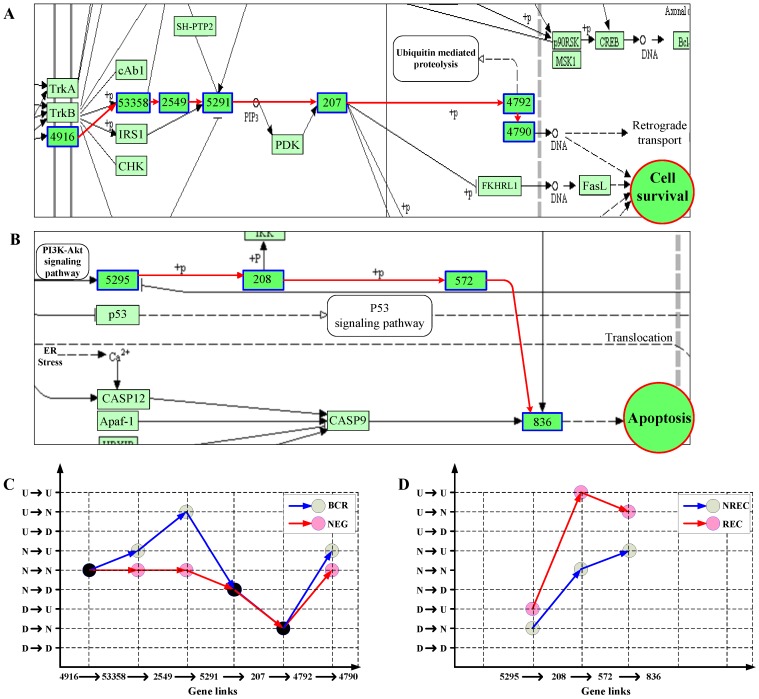
Key chains (**A**,**B**) and their principal patterns of state transitions (**C**,**D**) in two classes on the ALL (**A**,**C**) and liver (**B**,**D**) datasets. Genes involved are marked with blue-edge boxes and labeled with their Entrez IDs.

Biological information is necessary to propagate along pathway networks for the mediation and maintenance of the life of cells. Such signaling should be manifested in the state transition patterns of gene chains. We then recognized the principal patterns of pathways specific to each of the two classes for the ALL and liver datasets. For the ALL data, take neurotrophin signaling pathway as example and consider one of the two significant NF-κB-ending cascades, i.e., 4916 → 53358 → 2549 → 5291 → 207 → 4792 → 4790 (Entrez IDs), as shown in Figure 6A. Figure 6C compares the principal patterns of the cascade between the two ALL classes, BCR and NEG. From Figure 6C, we can clearly see that the two classes have two distinct state transition patterns with respective to the link cascade. Especially, in NEG, the consecutive D or N states along the path lead to the low expression of NF-κB, implying the suppression of cellular survival in NEG. We also noticed that signaling protein, GAB1 (Entrez ID: 2549), is mainly upregulated in BCR but not in NEG. This is in accordance with the experimental observation that GAB1 is tyrosine-phosphorylated in response to B cell antigen receptor engagement [36,37]. For the liver cancer data, similar results were also obtained. Figure 6B,D shows the result for one of three-link chain in differentially expressed hepatitis B pathway: 5295 → 208 → 572 → 863 (Entrez IDs), which is involved in the activation cascade of caspases responsible for apoptosis execution. From Figure 6B,D, it can be seen that the principal pattern in NREC, D → N → N → U, leads to overexpression of *CASP3* (Entrez ID: 863), and finally, activates the apoptosis of cell. This is consistent with the non-recurrence of liver cancer in NREC patients.

## 4. Discussion

Currently, most of current methods are based on counting DEGs, in which only the static information in pathways is considered. By contrast, the proposed method extracts signaling flows along a pathway by modeling gene links. As a result, the proposed method takes advantage of pathway topology, as well as characterizes dynamics of pathways based on MCM. One of advantages of the proposed method is that it allows for detecting abnormal state transitions along gene links or pathways in cancer for the purpose of DEP identification, as demonstrated in applications to two real-world cancer datasets (Figure 5 and Figure 6). For example, based on the liver cancer data, we find that a gene chain ending with *CASP3*, which inactivates *CASP3* and suppresses apoptosis of cells, is potentially associated with the recurrence of primary liver cancer patients. These will definitely help to gain deep insight into the molecular mechanisms of cancer. 

A comprehensive comparison of the classification power of gene links with those of random gene pairs confirmed the justification of DynSig in capturing the disparity of dynamics of pathways between classes (Figure 5). In particular, DynSig accurately called eight of the nine *BCR/ABL1*-involved pathways for the ALL dataset outperforming previous methods (Table 1). These initial results on gene links of length two genes are encouraging, and future work will extend DynSig to gene chains of length three or more genes, that provide a more objective and comprehensive understanding of signaling dynamics along pathways.

## 5. Conclusions

We have proposed a signaling dynamics-based approach, DynSig, for identifying differential pathways from high-throughput transcriptomics data analysis. The method takes emphasis on gene links, instead of gene nodes as usual, which facilitate the use of topological information of pathways in pathway analysis. Specifically, we first decompose the pathway networks into a set of gene links and introduce MCM to characterize and capture the dynamics of pathways. Finally, a new signaling dynamics-based statistic was derived to measure the disparity of pathways between different conditions of cells. Experimental results on simulation data and two real-world datasets, liver cancer and ALL datasets, demonstrated the effectiveness and efficiency of the proposed method. 

## Figures and Tables

**Table 1:** Performance comparison (%) of different methods on simulation data with cascade/complex pathway topology and *r* = 0.5.

Method	TPR	FPR	FNR	ACC	PPV	MCC	AUC
*ρ* = 0.3							
Our method	**56.40/48.30**	7.90/9.30	**43.60/51.70**	**74.25/69.50**	**88.51/85.84**	**52.32/42.66**	**87.19/85.09**
Global	0.00/0.00	**0.00/0.00**	100.00/100.00	50.00/50.00	NA/NA	NA/NA	46.50/47.95
LRpath	3.30/4.10	3.60/3.80	96.70/95.90	49.85/50.15	47.02/52.61	−0.94/0.90	50.01/51.70
TAPPA	5.80/7.30	3.40/2.70	94.20/92.70	51.20/52.30	61.96/72.08	5.47/10.29	58.37/63.05
Clipper	0.20/1.00	0.50/1.70	99.80/99.00	49.85/49.65	NA/32.33	NA/−3.38	46.11/48.71
DEGraph	1.80/2.20	2.00/2.20	98.20/97.80	49.90/50.00	46.67/47.33	−0.79/−0.31	46.74/48.05
*ρ* = 0.5							
Our method	**96.70/94.10**	5.80/9.10	**3.30/5.90**	**95.45/92.50**	**94.61/91.84**	**91.30/85.67**	**99.69/98.24**
Global	0.00/0.00	**0.00/0.00**	100.00/100.00	50.00/50.00	NA/NA	NA/NA	46.96/48.29
LRpath	2.40/4.00	4.70/4.70	97.60/96.00	48.85/49.65	32.59/46.33	−6.40/−1.67	49.87/50.26
TAPPA	8.80/4.50	2.40/1.60	91.20/95.50	53.20/51.45	77.12/72.25	13.48/8.11	70.07/63.95
Clipper	0.30/1.20	0.80/1.50	99.70/98.80	49.75/49.85	NA/48.00	NA/−0.71	47.43/47.30
DEGraph	1.80/2.40	2.20/2.10	98.20/97.60	49.80/50.15	NA/54.99	NA/1.00	46.93/47.28
*ρ* = 0.7							
Our method	**100.00/99.90**	6.40/8.60	**0.00/0.10**	**96.80/95.65**	**94.21/92.32**	**93.90/91.76**	**99.95/99.18**
Global	0.00/0.00	**0.00/0.00**	100.00/100.00	50.00/50.00	NA/NA	NA/NA	43.38/44.64
LRpath	3.30/3.50	5.30/5.60	96.70/96.50	49.00/48.95	36.85/36.46	−5.19/−5.40	49.94/48.92
TAPPA	19.30/2.20	0.60/0.40	80.70/97.80	59.35/50.90	97.20/NA	31.26/NA	87.69/63.11
Clipper	0.10/1.00	0.50/2.00	99.90/99.00	49.80/49.50	NA/24.88	NA/−4.92	44.28/44.61
DEGraph	1.50/1.80	2.40/2.40	98.50/98.20	49.55/49.70	32.64/35.48	−3.91/−3.01	44.98/47.14

The best results are marked in bold and NA means that no positives were reported. TPR: True positive rate; FPR: False positive rate; FNR: False negative rate; ACC: Average accuracy; PPV: Positive predictive value; MCC: Matthews correlation coefficient; AUC: Area under the curve.

**Table 2:** Identification results of the nine BCR/ABL1-involved pathways by our methods and previous methods for the ALL data.

Pathway	Our Method	Global Test	LRpath	TAPPA	Clipper	DEGraph
Axon guidance	√	√	☓	√	√	√
Cell cycle	√	√	√	☓	√	√
Chronic myeloid leukemia	√	√	☓	☓	√	√
ErbB signaling pathway	☓	√	☓	☓	☓	√
Neurotrophin signaling pathway	√	√	☓	☓	√	√
Pathogenic *Escherichia coli* infection	√	√	☓	☓	√	√
Pathways in cancer	√	√	☓	☓	☓	√
Shigellosis	√	√	☓	☓	√	☓
Viral myocarditis	√	☓	√	☓	√	☓

## Supplementary Materials

The following are available online at http://www.mdpi.com/2073-4425/9/7/323/s1, Figure S1: Comparison of changing pattern of AUCs among our method and five previous methods on simulation data. , Figure S2: Comparison of CPD curves of q-values among our and previous methods on the liver cancer (a) and ALL (b) data, Table S1: Performance Comparison of different methods on simulation DATA with CASCADE/Complex pathway topology under r = 0.6 and ρ = 0.3, 0.5, 0.7, Table S2: Performance Comparison of different methods on simulation DATA with CASCADE/Complex pathway topology under r = 0.7 and ρ = 0.3, 0.5, 0.7, Table S3: Performance Comparison of different methods on simulation DATA with CASCADE/Complex pathway topology under r = 0.8 and ρ = 0.3, 0.5, 0.7, Table S4: Differentially expressed pathways on liver cancer data, Table S5: Differentially expressed pathways on ALL data.
